# Sex Associations Between Air Pollution and Estimated Atherosclerotic Cardiovascular Disease Risk Determination

**DOI:** 10.3389/ijph.2023.1606328

**Published:** 2023-09-28

**Authors:** Alexandre Vallée

**Affiliations:** Department of Epidemiology and Public Health, Foch Hospital, Suresnes, France

**Keywords:** air pollution, atherosclerosis, cardiovascular risk, sex, public health

## Abstract

**Objective:** The purpose of this study was to investigate the sex correlations of particulate matters (PM_2.5_, PM_10_, PM_2.5–10_), NO_2_ and NOx with ASCVD risk in the UK Biobank population.

**Methods:** Among 285,045 participants, pollutants were assessed and correlations between ASCVD risk were stratified by sex and estimated using multiple linear and logistic regressions adjusted for length of time at residence, education, income, physical activity, Townsend deprivation, alcohol, smocking pack years, BMI and rural/urban zone.

**Results:** Males presented higher ASCVD risk than females (8.63% vs. 2.65%, *p* < 0.001). In males PM_2.5_, PM_10_, NO_2_, and NO_x_ each were associated with an increased ASCVD risk >7.5% in the adjusted logistic models, with ORs [95% CI] for a 10 μg/m^3^ increase were 2.17 [1.87–2.52], 1.15 [1.06–1.24], 1.06 [1.04–1.08] and 1.05 [1.04–1.06], respectively. In females, the ORs for a 10 μg/m^3^ increase were 1.55 [1.19–2.05], 1.22 [1.06–1.42], 1.07 [1.03–1.10], and 1.04 [1.02–1.05], respectively. No association was observed in both sexes between ASCVD risk and PM_2.5–10_.

**Conclusion:** Our findings may suggest the possible actions of air pollutants on ASCVD risk.

## Introduction

Continuous exposure to air pollution over an extended period poses a significant public health threat [1]. Alarmingly, a significant proportion of Europeans living in urban areas, up to 30%, are exposed to air pollution levels exceeding European air-quality standards [2]. Air pollution is recognized as one of the most significant yet often overlooked threats to human health [3–6]. The World Health Organization (WHO) estimates that air pollution is responsible for 7.6% of global mortality [7].

Recently, the 2019 European Society of Cardiology (ESC) guidelines emphasized the impact of air pollution on cardiovascular diseases [8]. Numerous studies have established a direct link between long-term exposure to air pollution and an increased risk of cardiovascular events, including myocardial infarction, stroke, and cardiovascular mortality [9, 10]. Furthermore, controlled environment studies have demonstrated that exposure to particulate matter induces systemic vascular responses, oxidative stress, endothelial dysfunction, and plaque formation [11].

Atherosclerotic cardiovascular disease (ASCVD) is a leading cause of mortality and contributes significantly to healthcare costs worldwide [12]. However, few investigations have focused on individual air pollutants, such as particulate matter (PM_2.5_, PM_10_), nitrogen oxide (NO_x_), and nitrogen dioxide (NO_2_), and their correlations with ASCVD risk [11, 13, 14].

Numerous investigations have shown sex differences for ASCVD risk and cardiovascular diseases [15, 16], including coronary heart disease [17], carotid atherosclerosis [18], hypertension [19], atrial fibrillation ([20], pp. 1994–2016), cardiometabolic disorders [21] and stroke [22].

Furthermore, sex differences in air pollution-related cardiovascular diseases have been highlighted [23] with an impact on circulatory and respiratory mortality [24]. But there has been limited research into the relationship between air pollution and ASCVD risk, and particularly concerning sex differences. This connection is still incomplete [23, 25]. Thus, the purpose of this study was to investigate, firstly, the association of particulate matters, nitrogen dioxide and nitrogen oxide with 10 year ASCVD risk in the overall population, and secondly, in sex-stratification analyses these associations, in the UK Biobank population.

## Methods

### UK Biobank Population

Between 2006 and 2010, the UK Biobank recruited a total of 502,478 individuals aged 38–73 years from 22 cities across the UK, representing approximately 5.5% of the overall UK population. The participants provided comprehensive information through questionnaires, computer-assisted interviews, and a variety of physical and functional measurements. Additionally, samples of blood, urine, and saliva were collected for further analysis [26]. Further details about the cohort’s protocol can be found in relevant literature [27, 28].

### Ethical Considerations

Prior to participation, all individuals provided electronic informed consent, and the UK Biobank obtained ethical approval from the North-West Multi-center Research Ethics Committee (MREC), which encompassed the entirety of the United Kingdom. The study adhered to the principles outlined in the Declaration of Helsinki and received approval from the Northwest—Haydock Research Ethics Committee (protocol code: 21/NW/0157, approval date: 21 June 2021). For details: [29].

### Study Population

**FIGURE 1:**
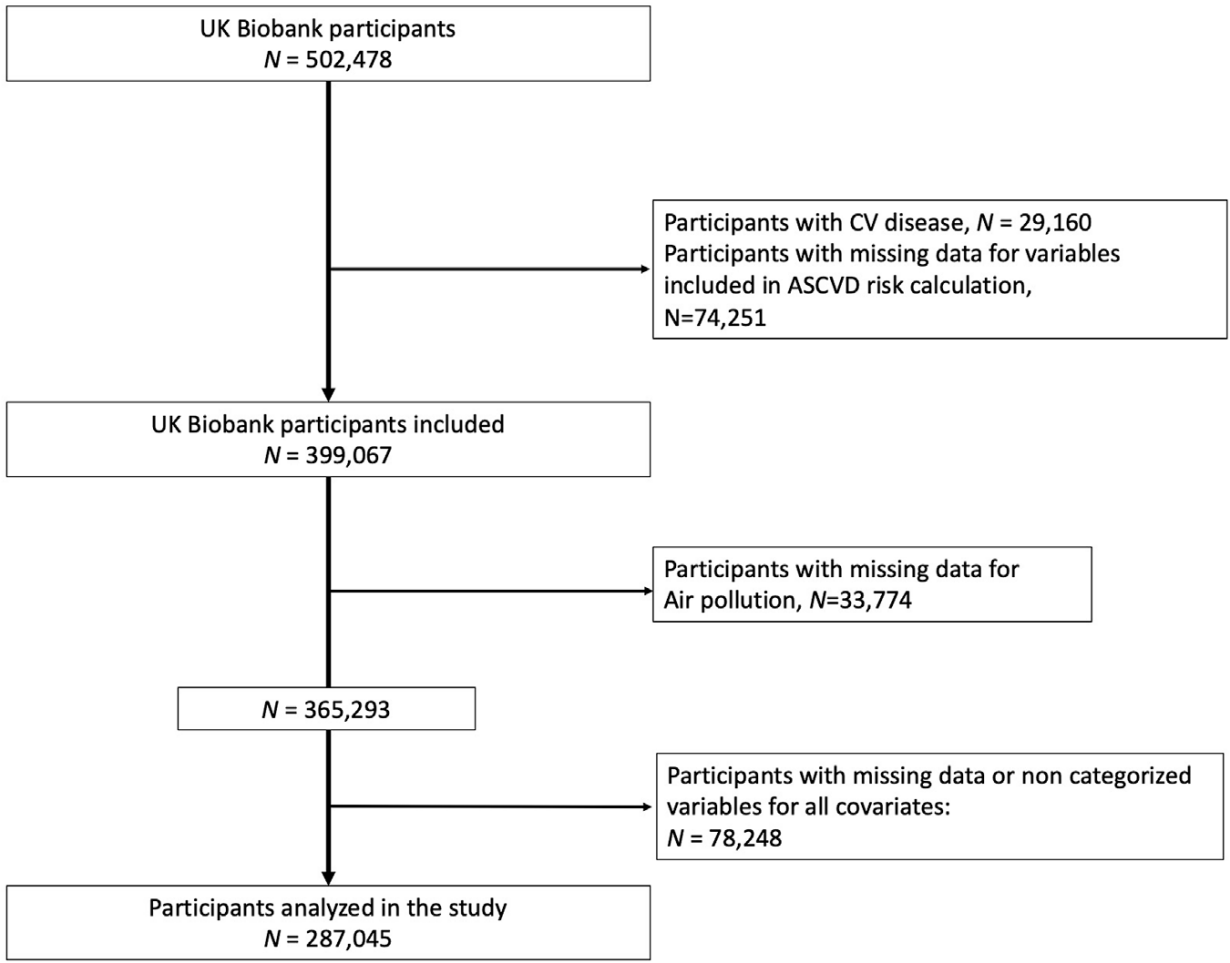
Flowchart (United Kingdom, 2021).

This study included a total of 399,067 participants from the UK Biobank who did not have missing data for ASCVD risk calculation and had no previous cardiovascular (CV) events. “CV diseases, including heart attack, angina, and stroke, were identified based on doctor diagnoses reported in the questionnaires” [30]. Moreover, this study also excluded 112,022 participants due to missing data for air pollutants (*N* = 33,774) and for other covariates (*N* = 78,248), resulting in a final analysis cohort of 287,045 participants (Figure 1).

### Estimated 10 Years ASCVD Risk

To estimate the 10 years ASCVD risk, the study utilized the Pooled Cohort Equations (PCE) model [31, 32]. The PCE enables the calculation of sex- and race-specific estimates of the 10-year risk of ASCVD for adults between the ages of 40 and 79. A PCE score of 7.5% or higher indicates a high risk of ASCVD, while a score below 7.5% suggests a lower risk [33–36].

### Air Pollution

The annual average concentrations of PM_2.5_, PM_10_, PM_2.5–10_, NO_2_, and NO_x_ were estimated using the Land Use Regression (LUR) model, which was developed as part of the European Study of Cohorts for Air Pollution Effects project [37, 38]. The LUR model incorporated geospatial predictor variables derived from a Geographic Information System, including factors such as traffic, land use, and topography. These variables were used to calculate the spatial variations in annual average air pollutant concentrations.

For this study, the residential addresses provided by participants during the baseline visit in the UK Biobank were utilized to link the air pollution exposures of each participant to their records. The exposure data for PM_2.5_, PM_10_, PM_2.5–10_, NO_2_, and NO_x_ were collected in 2010. In the case of NO_2_ and PM_10_, annual concentration data were available for multiple years (2005, 2006, 2007, and 2010 for NO_2_; 2007 and 2010 for PM_10_). The averaged values of NO_2_ and PM_10_ from these years were included in the analysis [39].

### Covariates

Blood pressure measurements, including systolic and diastolic blood pressure (SBP, DBP), were recorded twice using an automated BP device (Omron 705 IT electronic blood pressure monitor; OMRON Healthcare Europe B.V. Kruisweg 577 2132 NA Hoofddorp) at the assessment center [40, 41].

The determination of diabetes status was based on several criteria: receiving anti-diabetic medication, being diagnosed with diabetes by a doctor, or having a fasting glucose concentration ≥7 mmol/L [41, 42]. Medications were identified through the question: “Do you regularly take any of the following medications?”. Self-reported information on medication use was thus collected. Detailed information on biological parameters can be found in the UK Biobank protocol [43]. Body mass index (BMI) was calculated as weight (in kg) divided by height squared (in meters) and categorized as high (BMI > 30 kg/m^2^), moderate (BMI between 25 and 30 kg/m^2^), and low (less than 25 kg/m^2^) [44].

Education level was classified into three categories according to the International Standard Classification for Education (ISCED): high (college or university degree (20 years of education), NVQ or HND or HNC or equivalent [19 years of education)], moderate [O levels/GCSEs or equivalent (10 years of education), A/AS levels or equivalent (13 years of education), other professional qualifications such as nursing or teaching, etc. (15 years of education)], and low [none of the aforementioned (7 years of education)] [45]. Income level was defined as high (greater than £52,000 per year), moderate (between £18,000 and £51,999 per year), and low (less than £18,000 per year) [36]. Townsend deprivation index scores were calculated using data aggregated for the participants’ residential postcodes [46].

Current tobacco smokers were identified as participants who responded “yes, on most or all days” or “yes, only occasionally” to the question “do you smoke tobacco now.” Smoking pack-years were calculated as the average number of packs smoked per day multiplied by the total number of years of smoking in lifetime. In the UK Biobank, the number of years of smoking is calculated by subtracting the age of starting smoking from the age smoking was stopped (or age at inclusion for current smokers) [47, 48].

Alcohol drinker status was determined based on participants’ responses regarding their alcohol consumption: “current,” “past,” or “never” [35, 49].

Participants’ physical activity levels were evaluated using a revised version of the International Physical Activity Questionnaire (IPAQ) [50], which they completed on a tablet computer during the initial assessment. The self-reported physical activity data were analyzed using the approach developed by Bradbury et al. [51]. The total MET-hours per week were categorized as low (<10.0), moderate (10.0–49.9), and high (≥50 excess MET-hours/week) based on the IPAQ guidelines [52].

### Statistical Analysis

The characteristics of the study population were presented using median and 25th and 75th percentiles for continuous variables, and numbers and proportions for categorical variables. Group comparisons were conducted using either Student’s t-test or Mann-Whitney test for continuous variables, and Pearson’s χ^2^ test for categorical variables. The primary objective of the study was to examine the correlation between air pollutants and estimated 10 years ASCVD risk levels, as well as the risk of high cardiovascular (CV) risk (estimated 10 years ASCVD risk exceeding 7.5%). Multiple regression models were employed to analyze the relationship between air pollutants and estimated 10 years ASCVD risk. The results were expressed as beta coefficients (for each 10 μg/m^3^ increase) with 95% confidence intervals for the estimated 10 years ASCVD risk, and as odds ratios (OR) with 95% confidence intervals for the risk of exceeding 7.5% estimated 10 years ASCVD risk. The beta coefficients and OR were calculated per unit increase in 10 μg/m³ for air pollutants, and with the reference being the lowest quartile values of air pollutants (OR = 1.00). Firstly*,* the models were adjusted for various factors, including sex*,* length of time at residence, education, income level, physical activity, Townsend deprivation quintiles, alcohol consumption, smoking pack years, BMI categories, and rural/urban zone. Secondly, the models were stratified by sex. Analyses were stratified by sex through sex differences have been observed for ASCVD risk [15, 53], for air pollution impact on health [54, 55] and an interaction between sex and each air pollutants in this study (p for interaction, *p* < 0.001). Interactions were examined by including simultaneous sex, individual air pollutants and their interaction term. The adjustments were justified by their relationship with ASCVD risk and CV risk: education [56], income [30, 57], physical activity [58], smoking pack years [59], Townsend deprivation [30, 60] and BMI [61].

Multicollinearities between air pollutants have been investigated by spearman correlation coefficients. PM_2.5_, PM_10_, PM_2.5–10_, NO_2_ and NOX were considered independent in the different models due to their observed collinearities. Air pollutants were considered as continuous covariables and then, were categorized by quartiles (Q4 was the higher levels of air pollutants and Q1 the lowest quartile of levels). Non-linear relationship between air pollutants and CVD has been observed in previous studies [62, 63]. Thus, unadjusted non-linear splines of air pollutants with ASCVD risk were performed by polynomial quadratic regressions.

Statistics were performed using SAS software (version 9.4; SAS Institute, Carry, NC). A *p*-value < 0.05 was considered statistically significant.

## Results

**FIGURE 2:**
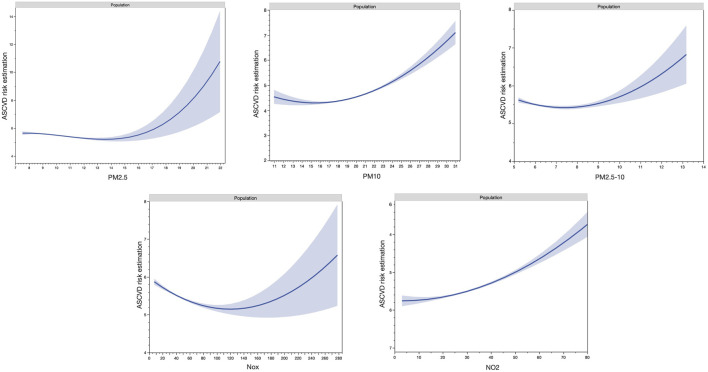
Non-linear splines of air pollutants with atherosclerotic cardiovascular disease risk (United Kingdom, 2021).

The characteristics of the overall participants are shown in Table 1. Males (*n* = 136,200, 47.4%) presented a higher mean estimated 10 years ASCVD risk compared to females (8.63% vs. 2.65%, *p* < 0.001), higher levels of high physical activity (23.49% vs. 19.39%, *p* < 0.001), of high educational level (37.68% vs. 35.35%, *p* < 0.001), of high income (30.60% vs. 24.82%, *p* < 0.001), of high BMI levels (23.75% vs. 21.91%, *p* < 0.001) but same proportion of rural residency (15.66% vs. 15.45%, *p* = 0.113) (Table 1). The mean (SD) concentrations of air pollutants were higher in males than in females, for PM_10_ (19.10 [18.00–20.35] μg/m^3^ vs. 19.14 [18.05–20.41] μg/m^3^, respectively, *p* < 0.001) and NO_2_ (27.80 [22.68–33.62] μg/m^3^ vs. 27.93 [22.77–33.79] μg/m^3^, respectively (*p* < 0.001) but not PM_2.5_, PM_2.5–10_ and NOx. The Spearman correlation coefficients among the five air pollutants are shown in Supplementary File S1. Splines of air pollutants with ASCVD risk are shown in Figure 2 and presented the non-linear relationships between air pollution and ASCVD risk.

**TABLE 1:** Characteristics of the study population (United Kingdom, 2021).

	Males *N* = 136,200		Females *N* = 150,845		*p*-value
Age (years) (mean, SD)	56.12	8.17	55.55	7.97	<0.001
Estimated 10 years ASCVD risk (%) (mean, SD)	8.63	6.68	2.65	3.01	<0.001
High level of ASCVD risk (>7.5%)	62,918	46.25%	9,089	6.03%	<0.001
Physical activity					<0.001
High	31,988	23.49%	29,247	19.39%	
Moderate	69,482	51.01%	80,058	53.07%	
Low	34,730	25.50%	41,540	27.54%	
BMI (kg/m^2^) (mean, SD)	27.65	4.11	26.83	5.06	<0.001
BMI					<0.001
High	32,348	23.75%	33,050	21.91%	
Moderate	68,236	50.10%	55,028	36.48%	
Low	35,616	26.15%	62,767	41.61%	
Education					<0.001
High	51,321	37.68%	53,324	35.35%	
Moderate	59,263	43.51%	70,769	46.92%	
Low	25,616	18.81%	26,752	17.73%	
Income					<0.001
High	41,684	30.60%	37,446	24.82%	
Moderate	70,323	51.63%	79,090	52.43%	
Low	24,193	17.76%	34,309	22.74%	
Quintiles Townsend deprivation					<0.001
Q1	27,477	20.17%	29,079	19.28%	
Q2	27,842	20.44%	30,194	20.02%	
Q3	27,326	20.06%	30,682	20.34%	
Q4	26,623	19.55%	31,020	20.56%	
Q5	26,932	19.77%	29,870	19.80%	
Antihypertensive medication	27,297	20.04%	22,570	14.96%	<0.001
Diabetes	9,606	7.06%	6,899	4.58%	<0.001
Black people	460	0.34%	752	0.50%	<0.001
Current alcohol drinkers	128,966	94.69%	138,880	92.07%	<0.001
Current smoking	15,865	11.65%	12,926	8.57%	<0.001
Smoking pack years	9.55	16.96	5.85	11.97	<0.001
HDL cholesterol (mmol/L) (mean, SD)	1.29	0.31	1.60	0.37	<0.001
Total cholesterol (mmol/L) (mean, SD)	5.58	1.08	5.88	1.10	<0.001
SBP (mmHg) (mean, SD)	138.91	15.84	126.71	17.41	<0.001
Rural population	21,333	15.66%	23,303	15.45%	0.113
Length of time at residence (years) (mean, SD)	16.59	12.16	16.64	11.97	0.221
PM_10_ (μg/m^3^) (median, IQR)	19.10	[18.00–20.35]	19.14	[18.05–20.41]	<0.001
NO_2_ (μg/m^3^) (median, IQR)	27.80	[22.68–33.62]	27.93	[22.77–33.79]	<0.001
PM_2.5_ (μg/m^3^) (median, IQR)	9.91	[9.26–10.53]	9.92	[9.27–10.55]	0.108
PM_2.5–10_ (μg/m^3^) (median, IQR)	6.10	[5.84–6.63]	6.10	[5.84–6.62]	0.107
NO_x_ (μg/m^3^)	41.80	[33.74–50.39]	42.04	[33.98–50.61]	0.096

When considering the WHO 2005 recommendations [64] for annual toxicity threshold 46.04% of males and 46.55% of females were over the threshold for PM_2.5_ and 30.90% and 31.78%, respectively for PM_10_, and 10.98% and 11.45%, respectively for NO_2_. However, when considering the 2021 WHO recommendations [65], nearly 100% of the population showed over toxicity thresholds (Table 2).

**TABLE 2:** Proportion of males and females exposed to toxicity thresholds of Particle Matter 2.5, Particle Matter 10 and Nitrogen dioxide according to World Health Organization 2005 and [65] classifications (United Kingdom, 2021).

	Males		Females	
	WHO 2005 (%)	WHO 2021 (%)	WHO 2005 (%)	WHO 2021 (%)
PM_2.5_	46.04	100.00	46.55	100.00
PM_10_	30.90	98.99	31.78	98.94
NO_2_	10.98	100.00	11.45	99.99

The associations between individual air pollutants and continuous ASCVD risk according to overall population and sex are shown in Table 3.

**TABLE 3:** Adjusted beta coefficients and 95% confidence interval for air pollution concentrations with continuous atherosclerotic cardiovascular disease risk (United Kingdom, 2021).

Overall population					
	Q1	Q2	Q3	Q4	Continuous
PM_2.5_	Ref.	−0.033 (−0.064; 0.001)	0.067 (0.035; 0.099)	0.169 (0.134; 0.203)	1.242 (1.038; 1.446)
PM_10_	Ref.	−0.020 (−0.052; 0.011)	0.073 (0.041; 0.104)	0.073 (0.039; 0.0106)	0.320 (0.214; 0.426)
PM_2.5–10_	Ref.	0.016 (−0.015; 0.046)	0.005 (−0.026; 0.036)	0.022 (−0.009; 0.053)	0.296 (0.094; 0.497)
NO_2_	Ref.	−0.006 (−0.038; 0.027)	0.151 (0.118; 0.183)	0.111 (0.076; 0.147)	0.105 (0.079; 0.129)
NO_X_	Ref.	−0.065 (−0.096; 0.005)	0.113 (0.081; 0.145)	0.185 (0.150; 0.219)	0.078 (0.065; 0.093)

Males					
	Q1	Q2	Q3	Q4	Continuous
PM_2.5_	Ref.	−0.041 (−0.100; 0.019)	0.121 (0.061; 0.181)	0.227 (0.163; 0.291)	1.776 (1.392; 2.159)
PM_10_	Ref.	−0.037 (−0.096; 0.022)	0.085 (0.026; 0.144)	0.99 (0.036; 0.162)	0.434 (0.233; 0.635)
PM_2.5–10_	Ref.	0.024 (−0.034; 0.083)	0.014 (−0.044; 0.072)	0.009 (−0.049; 0.068)	0.323 (−0.055; 0.702)
NO_2_	Ref.	0.003 (−0.058; 0.063)	0.209 (0.148; 0.271)	0.168 (0.101; 0.234)	0.158 (0.111; 0.205)
NO_X_	Ref.	−0.100 (−0.181; 0.010)	0.186 (0.126; 0.246)	0.253 (0.188; 0.319)	0.111 (0.085; 0.136)

Females					
PM_2.5_	Ref.	−0.019 (−0.045; 0.007)	0.022 (−0.003; 0.049)	0.096 (0.067; 0.124)	0.617 (0.447; 0.788)
PM_10_	Ref.	−0.002 (−0.028; 0.024)	0.056 (0.029; 0.082)	0.036 (0.008; 0.064)	0.160 (0.072; 0.248)
PM_2.5–10_	Ref.	0.013 (−0.013; 0.039)	−0.008 (−0.029; 0.024)	0.027 (0.001; 0.053)	0.221 (0.053; 0.389)
NO_2_	Ref.	−0.019 (0.046; 0.007)	0.091 (0.064; 0.118)	0.065 (0.036; 0.095)	0.049 (0.027; 0.069)
NO_X_	Ref.	−0.036 (−0.062; 0.009)	0.045 (0.018; 0.071)	0.111 (0.083; 0.140)	0.041 (0.029; 0.052)

Models were adjusted for length of time at residence, education, income level, physical activity, Townsend deprivation quintiles, alcohol consumption, smoking pack years, BMI categories, and rural/urban zone.

Air pollutants were considered as continuous covariables and then, were categorized by quartiles (Q4 was the higher levels of air pollutants and Q1 the lowest quartile of levels).

Beta coefficient of continuous variable of air pollutants were expressed by 10 μg/m^3^.

In overall population, PM_2.5_, PM_10_, NO_2_, and NO_x_ each were associated with an increased continuous ASCVD risk in the adjusted linear models. No significant correlation was observed in males in the adjusted model between increased ASCVD risk and PM_2.5–10_, except when considering PM_2.5–10_ as a continuous variable.

In males, PM_2.5_, PM_10_, NO_2_, and NO_x_ each were associated with an increased continuous ASCVD risk in the adjusted linear models. The beta coefficients (95% CI) of increase in continuous ASCVD risk for a 10 μg/m^3^ increase in PM_2.5_, PM_10_, NO_2_, and NO_x_ were 1.776 (1.392; 2.159), 0.434 (0.233; 0.635), 0.158 (0.111; 0.205) and 0.111 (0.085; 0.136), respectively. No significant correlation was observed in males in the adjusted model between increased ASCVD risk and PM_2.5–10_ (Table 3).

Among females, both PM_2.5_, PM_10_, PM_2.5–10_, NO_2_, and NO_x_ were associated with an increased continuous ASCVD risk in the adjusted linear models. The beta coefficients (95% CI) of increase in continuous ASCVD risk for a 10 μg/m^3^ increase in PM_2.5_, PM_10_, PM_2.5–10_, NO_2_, and NO_x_ were 0.617 (0.447; 0.788), 0.160 (0.072; 0.248), 0.221 (0.053; 0.389), 0.049 (0.027; 0.069), and 0.041 (0.029; 0.052), respectively (Table 3).

When considering ASCVD risk >7.5%, in overall population, PM_2.5_, PM_2.5–10_, PM_10_, NO_2_, and NO_x_ were all associated with an increased ASCVD risk >7.5% in the adjusted logistic models (Table 4).

**TABLE 4:** Adjusted Odds ratio and 95% confidence interval for air pollution concentrations with atherosclerotic cardiovascular disease risk >7.5% (United Kingdom, 2021).

Overall population					
	Q1	Q2	Q3	Q4	Continuous
PM_2.5_	Ref.	1.11 [1.07–1.15]	1.19 [1.14–1.23]	1.24 [1.19–1.28]	2.05 [1.80–2.34]
PM_10_	Ref.	1.04 [1.01–1.07]	1.09 [1.06–1.13]	1.09 [1.06–1.13]	1.18 [1.09–1.26]
PM_2.5–10_	Ref.	1.03 [1.01–1.07]	1.04 [1.01–1.08]	1.04 [1.01–1.08]	1.11 [1.01–1.21]
NO_2_	Ref.	1.14 [1.10–1.18]	1.23 [1.18–1.28]	1.23 [1.18–1.18]	1.06 [1.04–1.108]
NO_X_	Ref.	1.10 [1.07–1.14]	1.22 [1.18–1.27]	1.27 [1.22–1.32]	1.05 [1.03–1.06]

Males					
	Q1	Q2	Q3	Q4	Continuous
PM_2.5_	Ref.	1.12 [1.08–1.16]	1.20 [1.16–1.25]	1.26 [1.20–1.32]	2.17 [1.87–2.52]
PM_10_	Ref.	1.04 [1.01–1.08]	1.09 [1.05–1.13]	1.08 [1.04–1.13]	1.15 [1.06–1.24]
PM_2.5–10_	Ref.	1.04 [0.99–1.08]	1.03 [0.99–1.07]	1.03 [0.99–1.07]	1.12 [0.97–1.29]
NO_2_	Ref.	1.15 [1.11–1.20]	1.23 [1.18–1.29]	1.23 [1.17–1.29]	1.06 [1.04–1.08]
NO_X_	Ref.	1.12 [1.07–1.16]	1.25 [1.20–1.30]	1.29 [1.23–1.35]	1.05 [1.04–1.06]

Females					
PM_2.5_	Ref.	1.07 [0.99–1.15]	1.12 [1.04–1.21]	1.14 [1.06–1.24]	1.55 [1.19–2.05]
PM_10_	Ref.	1.03 [0.96–1.11]	1.10 [1.02–1.18]	1.11 [1.03–1.20]	1.22 [1.06–1.42]
PM_2.5–10_	Ref.	1.02 [0.95–1.09]	1.05 [0.97–1.12]	1.07 [0.99–1.14]	1.24 [0.95–1.62]
NO_2_	Ref.	1.09 [1.00–1.17]	1.21 [1.12–1.31]	1.20 [1.10–1.31]	1.07 [1.03–1.10]
NO_X_	Ref.	1.06 [0.99–1.14]	1.12 [1.04–1.21]	1.18 [1.09–1.28]	1.04 [1.02–1.05]

Models were adjusted for length of time at residence, education, income level, physical activity, Townsend deprivation quintiles, alcohol consumption, smoking pack years, BMI categories, and rural/urban zone.

Air pollutants were considered as continuous covariables and then, were categorized by quartiles (Q4 was the higher levels of air pollutants and Q1 the lowest quartile of levels).

Odds ratio of continuous variable of air pollutants were expressed by 10 μg/m^3^.

In males PM_2.5_, PM_10_, NO_2_, and NO_x_ each were associated with an increased ASCVD risk>7.5% in the adjusted logistic models. The ORs [95% CI] of increase in continuous ASCVD risk for a 10 μg/m^3^ increase in PM_2.5_, PM_10_, NO_2_, and NO_x_ were 2.17 [1.87–2.52], 1.15 [1.06–1.24], 1.06 [1.04–1.08] and 1.05 [1.04–1.06], respectively. No significant association was observed in males in the adjusted model between increased ASCVD risk and PM_2.5–10_ (Table 4).

Among females, both PM_2.5_, PM_10_, NO_2_, and NO_x_ were associated with an increased ASCVD risk>7.5% in the adjusted logistic models. The ORs (95% CI) of increase in continuous ASCVD risk for a 10 μg/m^3^ increase in PM_2.5_, PM_10_, NO_2_, and NO_x_ were 1.55 [1.19–2.05], 1.22 [1.06–1.42], 1.07 [1.03–1.10], and 1.04 [1.02–1.05], respectively. No significant association was observed in males in the adjusted model between increased ASCVD risk and PM_2.5–10_ (Table 4).

For both sexes, when considering the higher quartile groups compared to the lowest quartile, similar results were expressed (Tables 3, 4). When considering the ASCVD risk >7.5%, in males PM_2.5_, PM_10_, NO_2_, and NO_x_ each were associated with an increased ASCVD risk >7.5% in the adjusted logistic models. The ORs [95% CI] for Q4 vs. Q1 were for PM_2.5_, PM_10_, NO_2_, and NO_x_, 1.26 [1.20–1.32], 1.08 [1.04–1.13], 1.23 [1.17–1.29], and 1.29 [1.23–1.35], respectively. And for females, the ORs [95% CI] for Q4 vs. Q1 were for PM_2.5_, PM_10_, NO_2_, and NO_x_, 1.12 [1.04–1.21], 1.10 [1.02–1.18], 1.21 [1.12–1.31] and 1.12 [1.04–1.21], respectively (Table 4). No significant association between PM_2.5–10_ and ASCVD risk was observed in both sexes.

### Sensitive Analysis

When adding the “time of enrollment” in analyses, similar results were observed (Supplementary File S2).

## Discussion

The primary finding of this study revealed a significant association between each individual air pollutants and an elevated risk of ASCVD in both males and females. Few sex differences were observed between individual air pollutants and ASCVD risk in this study. These results are consistent with previous studies in UK Biobank cohort [25, 39, 66–68].

### Sex Differences

In 2022, a meta-analysis shown that CV events and mortality were increased by short exposure to PM_2.5_, in same average rate in both sexes [69]. But, when considering the long-term exposure to PM_2.5_ a sex differences was observed for ischemic heart disease [25]. Nevertheless, it remains controversial whether substantial differences would exist in air pollution with CV events between sexes. Numerous studies reported similar risk between sexes [70–72], while others reported differences in sexes [73, 74]. To date, no biological mechanism may explain the plausible differences observed in studies.

While, no differences in risk was observed between sexes, accurate health risk assessment remains essential to delivery optimal preventive medical care, and it is no longer acceptable to use a one-size-fits-all model of cardiovascular risk stratification which ignores sex differences [75]. Many tools or equations of cardiovascular risk assessment widely recommended by guidelines were developed based on sex-specific models along with different effect estimations even for the same risk factor [33, 76]. In forthcoming research concerning the interplay between air pollution and cardiovascular wellbeing, it is advisable to consistently present outcomes specific to sex. In the context of preventing and treating ischemic heart disease (IHD), data from both the US and China revealed a notable trend. It indicated that women were at a lower likelihood of receiving accurate diagnoses and receiving preventative care compared to men. This tendency might be connected to a perception of reduced risk among clinicians and patients towards women [77, 78].

### Epidemiological Evidence

The findings of this study are consistent with prior studies examining the connection between air pollution and ASCVD risk [14, 79], heart failure [39], and ischemic heart disease [80]. Furthermore, air pollution has been associated with various other cardiovascular diseases, including cardiac arrhythmias and arrest, cerebrovascular disease, and venous thromboembolism [81, 82]. A study estimated that air pollution contributes to a global excess mortality of 8.8 million annually, resulting in a reduction of 2.9 years in life expectancy [83]. However, limited research has focused on different risk factors for ASCVD, such as blood lipid levels, hypertension, and diabetes [84–86].

Epidemiological studies have shown an association between prolonged exposure to PM_2.5_ and atherosclerosis, as measured by various indicators, such as carotid intima-media thickness, coronary, aortic calcifications, and ankle-brachial index [87]. Reduction in PM_2.5_ concentration has been associated to a decrease in the progression of intima-media carotid thickness [88]. The authors of the meta-analysis published in 2014 have found that prolonged exposure to particulate matter was associated with an increased incidence of coronary events [9]. Specifically, for every 5 μg/m^3^ increase in PM_2.5_ exposure, there was a 13% rise in non-fatal acute coronary events, and for every 10 μg/m^3^ increase in PM_10_ exposure, there was a 12% increase in coronary events. No relationship was found with other pollutants [9]. Another meta-analysis in 2015 demonstrated that PM_2.5_ and NO_2_ were significantly associated with diabetes [89]. Additionally, an increase in PM_2.5_ concentration by 10 μg/m^3^ has been linked to a 1–3 mmHg increase in systolic blood pressure a few days after exposure [90].

Moreover, the composition of PM stands as a pivotal aspect to deliberate, as certain discoveries highlight the heightened cardiovascular harm attributed to carbon-based particles originating from combustion-related sources like road traffic, fossil fuel usage, and wood combustion [91]. These combustion sources also contribute predominantly to the emission of nitrogen dioxide (NO_2_). A comprehensive analysis of the cardiovascular repercussions stemming from prolonged exposure to NO_2_ demonstrated a 13% surge in cardiovascular-related mortality following a mere 10 μg/m^3^ rise in annual NO_2_ concentrations [92]. The impact of ozone appears to exhibit a lesser magnitude, as some studies on long-term exposure imply a minor upswing in causes of cardiopulmonary mortality. Notably, this effect was only observable during warmer seasons and not in a year-round assessment. Numerous investigations have emphasized a robust link between extended exposure to air pollution and instances of acute myocardial infarction. In an expansive prospective study carried out across Europe, incremental yearly elevations of 10 μg/m^3^ in PM_10_ and 5 μg/m^3^ in PM_2.5_ were correlated with amplified risks of myocardial infarction, reaching 12% and 13% correspondingly [9].

### Pathophysiological Mechanisms

Exposure to PM, particularly exposure to the polycyclic aromatic hydrocarbons present on the surface of PM originating from traffic, displayed a correlation with elevated levels of 8-hydroxy-2′-deoxyguanosine [93]. This biomarker is recognized for its reliability in indicating oxidative DNA damage in individuals subjected to such exposure, including individuals like traffic policemen, bus drivers, and garage workers [94]. Similarly, the occurrence of etheno-DNA adducts was double in diesel engine workers when compared to their non-diesel-exposed counterparts [95]. Various research studies have reported links between air pollution exposure and heightened plasma levels of oxidized low-density lipoprotein, homocysteine, as well as inflammatory markers and fibrinogen [96]. More recently, escalated concentrations of intercellular adhesion molecule-1 and vascular cell adhesion molecule-1 have been tied to exposure to air pollution [97]. The impact of air pollution exposure might facilitate the activation of inflammatory genes, as evidenced by studies revealing upregulation of antioxidant genes and a decline in overall DNA methylation [97]. Multiple investigations have affirmed that air pollution exposure triggers a robust oxidative stress response upon PM entry into the lungs. However, this pulmonary oxidative reaction is intensified through the activation of various enzymatic pathways, ultimately culminating in a systemic vascular oxidative stress reaction. After incubating endothelial cell cultures with serum obtained from voluntarily exposed subjects, the production of superoxide anions (reactive oxygen species [ROS]) was observed. This production followed a dose-response pattern directly linked to the quantity of inhaled PM_2.5_ [98]. The predominant role of ROS production was further confirmed by *in vitro* studies, revealing that superoxide dismutase reverses the adverse vascular effects subsequent to diesel exhaust exposure [99]. Studies have shown that the oxidative stress response was predominantly linked to the surface compounds coating diesel particles, including transition metals, polycyclic aromatic hydrocarbons, and quinones [99]. While oxidative stress primarily associates with particles, gaseous components such as NO_2_ are also contributors to ROS generation, forming peroxynitrite [93].

Exposure to diesel exhaust, in contrast to filtered air, resulted in impaired endothelium-dependent vasodilation and diminished endothelial NO bioavailability [100]. Endothelial dysfunction stands as an early indicator of atherosclerosis [101], with some initial functional consequences evident across various vascular beds. A decrease in patients ischemic threshold was observable, suggesting a detrimental influence of air pollution on myocardial blood flow regulation [102]. Air pollution favored the production of oxidized low-density lipoprotein and the release of other highly oxidized phospholipids [101]. These proatherogenic molecules permeated the subendothelial space, triggering the activation of endothelial cells. This activation was marked by the release of proinflammatory adhesion molecules, such as vascular cell adhesion molecule-1 and monocyte chemotactic protein-1 [101]. Furthermore, air pollution was linked to the impairment of antiatherogenic molecules like high-density lipoprotein [103]. A reduction in the antioxidant capacity of high-density lipoprotein was also observed in an animal study, with ultra-fine particles exhibiting a more pronounced effect compared to PM_2.5_ [101]. Consequently, exposure to air pollution, such as PM and NO_2_ [104], triggered vascular inflammation, accumulation of lipids in foam cells, and progression of arterial plaque, leading to increase the ASCVD risk [103].

### Limitations

The study’s primary strength is its large sample size, which is drawn from the UK Biobank cohort. However, the cross-sectional design of the study limits the establishment of causal relationships and precludes determining reverse causation. Although the study had a response rate of only 5.5%, the robust sample size and high internal validity minimize the likelihood of participant bias influencing the observed associations [105, 106]. The generalizability of the study’s results to other age groups or ethnic populations may be limited since it focused exclusively on middle-aged participants from the UK. Nevertheless, the UK Biobank employed standardized protocols for data collection, ensuring replicability across all participants.

Several limitations should be acknowledged. Firstly, socio-economic data, medical history, and comorbidities were collected through self-reported questionnaires or physician assertion, which introduces the potential for bias. Additionally, the study’s temporal scope is restricted since data collection occurred only between 2006 and 2010. This study estimated the weights (regression coefficients) of air pollutants by treating each as a continuous variable. However, air pollutants were not linearly associated with ASCVD risk (Figure 2). Although examining non-linearity relations with the construction of quartiles of air pollutant may provide more information, the construction of the quartiles would not be sufficient. It is important to note that air pollution exposure may be either overestimated or underestimated, as the study did not gather data on pollution exposure in work environments, and only a single measurement of air pollution was available. The lack of address mobility is a major limitation in this study for the investigation of the relationship between air pollution exposure and ASCVD risk. Due to the high number of participants excluded due to missing data, the results observed should not be representative of the entire UK Biobank cohort. However, even though this study primarily focused on a specific population, it can still provide valuable insights as a reference when examining populations in other regions. Moreover, the results may not generalize to other populations, given that most participants in the UK Biobank were of European descent.

### Conclusion

This study provides evidence that exposure to air pollutants, such as PM_2.5_, PM_10_, PM_2.5–10_, NO_2_ and NOx, throughout the year is associated with an increased risk of ASCVD in both males and females. These findings suggest the potential effects of air pollutants on ASCVD risk. They also highlight the need for implementing appropriate preventive health policies for populations affected by air pollution. Given the link between air pollution and ASCVD risk, it is crucial to gather information on individuals residing in polluted and urban areas or during periods of elevated air pollution levels.

## Data Availability

The data that support the findings of this study are available on request from the corresponding author. The data are not publicly available due to privacy or ethical restrictions.
