# Supplementary material for: Sex Associations Between Air Pollution and Estimated Atherosclerotic Cardiovascular Disease Risk Determination
Source: Int J Public Health. 2023 Sep 28;68:1606328. doi: 10.3389/ijph.2023.1606328 (PMC10569126; doi:10.3389/ijph.2023.1606328)
Supplement: Supplementary file 1 [file DataSheet2.docx]

**Supplemental file 2**

**Sensitive analyses with adding as adjustment the time of enrollment in multivariable analyses.**

In overall population, time of enrollment was:

| year | Number and percentage |
| --- | --- |
| 2006 | 2 (0.00%) |
| 2007 | 22,564 (7.86%) |
| 2008 | 103,742 (36.14%) |
| 2009 | 105,067 (36.60%) |
| 2010 | 55,560 (19.39%) |

Supplemental table 1: Adjusted beta coefficients and 95% confidence interval for air pollution concentrations with continuous ASCVD risk. Air pollutants were considered as continuous covariables and then, were categorized by quartiles (Q4 was the higher levels of air pollutants and Q1 the lowest quartile of levels).

| **OVERALL population** | | | | | |
| --- | --- | --- | --- | --- | --- |
|  | Q1 | Q2 | Q3 | Q4 | Continuous |
| **PM2.5** | Ref. | -0.028 (-0.059 ; 0.003) | 0.069 (0.037 ; 0.101) | 0.158 (0.124 ; 0.193) | 1.185 (0.979 ; 1.391) |
| **PM10** | Ref. | -0.025 (-0.056 ; 0.006) | 0.062 (0.031 ; 0.094) | 0.094 (0.061 ; 0.129) | 0.383 (0.276 ; 0.490) |
| **PM2.5-10** | Ref. | -0.028 (-0.059 ; 0.003) | 0.069 (0.037 ; 0.101) | 0.158 (0.124 ; 0.193) | 0.319 (0.118 ; 0.521) |
| **NO2** | Ref. | -0.014 (-0.046 ; 0.018) | 0.139 (0.107 ; 0.173) | 0.135 (0.098 ; 0.171) | 0.119 (0.094 ; 0.145) |
| **NOX** | Ref. | -0.062 (-0.093 ; 0.029) | 0.115 (0.083 ; 0.147) | 0.178 (0.143 ; 0.213) | 0.0767 (0.063 ; 0.090) |
| **MALES** | | | | | |
|  |  |  |  |  |  |
|  | Q1 | Q2 | Q3 | Q4 | Continuous |
| **PM2.5** | Ref. | -0.037 (-0.098 ; 0.022) | 0.124 (0.064 ; 0.184) | 0.221 (0.156 ; 0.285) | 1.746 (1.361 ; 2.131) |
| **PM10** | Ref. | -0.043 (-0.103 ; 0.016) | 0.072 (0.013 ; 0.132) | 0.121 (0.058 ; 0.185) | 0.494 (0.292 ; 0.695) |
| **PM2.5-10** | Ref. | 0.019 (-0.040 ; 0.079) | 0.022 (-0.037 ; 0.081) | 0.012 (-0.047 ; 0.071) | 0.347 (-0.031 ; 0.727) |
| **NO2** | Ref. | -0.005 (-0.065 ; 0.055) | 0.199 (0.138 ; 0.261) | 0.186 (0.117 ; 0.254) | 0.169 (0.122 ; 0.217) |
| **NOX** | Ref. | -0.097 (-0.157 ; 0.037) | 0.187 (0.127 ; 0.247) | 0.247 (0.181 ; 0.312) | 0.109 (0.083 ; 0.134) |
| **FEMALES** | | | | | |
| **PM2.5** | Ref. | -0.014 (-0.040 ; 0.012) | 0.024 (-0.002; 0.051) | 0.086 (0.057 ; 0.114) | 0.553 (0.381 ; 0.726) |
| **PM10** | Ref. | -0.004 (-0.031 ; 0.021) | 0.048 (0.022 ; 0.082) | 0.054 (0.025 ; 0.082) | 0.214 (0.125 ; 0.303) |
| **PM2.5-10** | Ref. | 0.008 (-0.018; 0.034) | 0.006 (-0.019 ; 0.032) | 0.028 (0.002 ; 0.055) | 0.241 (0.073 ; 0.409) |
| **NO2** | Ref. | -0.027 (0.053 ; 0.001) | 0.081 (0.054 ; 0.109) | 0.087 (0.057 ; 0.118) | 0.062 (0.041 ; 0.083) |
| **NOX** | Ref. | -0.033 (-0.059 ; 0.006) | 0.046 (0.020 ; 0.073) | 0.105 (0.077 ; 0.135) | 0.039 (0.027 ; 0.050) |

Models were adjusted for length of time at residence, education, income level, physical activity, Townsend deprivation quintiles, alcohol consumption, smoking pack years, BMI categories, and rural/urban zone and time of enrollment.

Supplemental table 2: Adjusted Odd ratios and 95% confidence interval for air pollution concentrations with ASCVD risk>7.5%. Air pollutants were considered as continuous covariables and then, were categorized by quartiles (Q4 was the higher levels of air pollutants and Q1 the lowest quartile of levels).

| **OVERALL population** | | | | | |
| --- | --- | --- | --- | --- | --- |
|  | Q1 | Q2 | Q3 | Q4 | Continuous |
| **PM2.5** | Ref. | 1.11 [1.07 – 1.14] | 1.19 [1.14 – 1.23] | 1.23 [1.18 – 1.27] | 1.98 [1.73 – 2.26] |
| **PM10** | Ref. | 1.04 [1.01 – 1.07] | 1.09 [1.05 – 1.13] | 1.11 [1.07 – 1.15] | 1.21 [1.13 – 1.30] |
| **PM2.5-10** | Ref. | 1.04 [1.01 – 1.07] | 1.05 [1.01 – 1.08] | 1.05 [1.01 – 1.08] | 1.17 [1.03 - 1.32] |
| **NO2** | Ref. | 1.13 [1.09 – 1.18] | 1.22 [1.18 – 1.27] | 1.25 [1.19 – 1.30] | 1.07 [1.06 – 1.09] |
| **NOX** | Ref. | 1.11 [1.07 – 1.15] | 1.22 [1.18 – 1.27] | 1.26 [1.18 – 1.31] | 1.05 [1.04 – 1.06] |
| **MALES** | | | | | |
|  | Q1 | Q2 | Q3 | Q4 | Continuous |
| **PM2.5** | Ref. | 1.12 [1.08 – 1.16] | 1.20 [1.16 – 1.25] | 1.25 [1.20 – 1.30] | 2.10 [1.81 – 2.44] |
| **PM10** | Ref. | 1.04 [1.01 – 1.08] | 1.08 [1.04 – 1.12] | 1.10 [1.06 – 1.15] | 1.19 [1.10 – 1.28] |
| **PM2.5-10** | Ref. | 1.04 [0.99 – 1.08] | 1.03 [0.99 – 1.07] | 1.04 [0.99 – 1.07] | 1.13 [0.98 – 1.31] |
| **NO2** | Ref. | 1.15 [1.10 – 1.20] | 1.23 [1.17 – 1.28] | 1.24 [1.19 – 1.30] | 1.07 [1.05 – 1.09] |
| **NOX** | Ref. | 1.12 [1.07 – 1.16] | 1.25 [1.20 – 1.30] | 1.28 [1.22 – 1.34] | 1.05 [1.04 – 1.06] |
| **FEMALES** | | | | | |
| **PM2.5** | Ref. | 1.07 [0.99 – 1.16­] | 1.12 [1.04 – 1.21] | 1.13 [1.05 – 1.23] | 1.48 [1.13 – 2.42] |
| **PM10** | Ref. | 1.04 [0.97 – 1.11] | 1.10 [1.03 – 1.18] | 1.13 [1.05 – 1.22] | 1.27 [1.09 – 1.47] |
| **PM2.5-10** | Ref. | 1.02 [0.95 – 1.09] | 1.05 [0.98 – 1.13] | 1.07 [1.00 – 1.15] | 1.25 [0.96 – 1.64] |
| **NO2** | Ref. | 1.09 [1.01 – 1.18] | 1.21 [1.12 – 1.31] | 1.22 [1.12 – 1.33] | 1.08 [1.03 – 1.11] |
| **NOX** | Ref. | 1.06 [0.99 – 1.14] | 1.12 [1.04 – 1.21] | 1.17 [1.08 – 1.27] | 1.04 [1.02 – 1.05] |

Models were adjusted for length of time at residence, education, income level, physical activity, Townsend deprivation quintiles, alcohol consumption, smoking pack years, BMI categories, and rural/urban zone and time of enrollment.
