# Supplementary material for: Sex Associations Between Air Pollution and Estimated Atherosclerotic Cardiovascular Disease Risk Determination
Source: Int J Public Health. 2023 Sep 28;68:1606328. doi: 10.3389/ijph.2023.1606328 (PMC10569126; doi:10.3389/ijph.2023.1606328)
Supplement: Supplementary file 2 [file DataSheet1.docx]

Supplemental file 1: Coefficients of collinearity between air pollutants according to sex

| **Males** | **PM_10_** | **NO2** | **PM_2.5_** | **PM_2.5-10_** | **NOx** |
| --- | --- | --- | --- | --- | --- |
| **PM_10_** |  | 0.8006 | 0.6483 | 0.5276 | 0.6493 |
| **NO2** | 0.8006 |  | 0.7385 | 0.1777 | 0.7499 |
| **PM_2.5_** | 0.6483 | 0.7385 |  | 0.2187 | 0.8496 |
| **PM_2.5-10_** | 0.5276 | 0.1777 | 0.2187 |  | 0.2380 |
| **NOx** | 0.6493 | 0.7499 | 0.8496 | 0.2380 |  |
|  | | | | | |
| **Females** | **PM_10_** | **NO2** | **PM_2.5_** | **PM_2.5-10_** | **NOx** |
| **PM_10_** |  | 0.8051 | 0.6428 | 0.5205 | 0.6482 |
| **NO2** | 0.8051 |  | 0.7302 | 0.1771 | 0.7455 |
| **PM_2.5_** | 0.6428 | 0.7302 |  | 0.2129 | 0.8514 |
| **PM_2.5-10_** | 0.5205 | 0.1771 | 0.2129 |  | 0.2315 |
| **NOx** | 0.6482 | 0.7455 | 0.8514 | 0.2315 |  |
